# Influence of Surface Treatment on Steel Adhesive Joints Strength—Varnish Coats

**DOI:** 10.3390/ma14226938

**Published:** 2021-11-16

**Authors:** Anna Rudawska, Izabela Miturska-Barańska, Elżbieta Doluk

**Affiliations:** Department of Production Engineering, Faculty of Mechanical Engineering, Lublin University of Technology, Nadbystrzycka 36 St., 20-618 Lublin, Poland; i.miturska@pollub.pl (I.M.-B.); e.doluk@pollub.pl (E.D.)

**Keywords:** surface treatment method, varnish coat, adhesive joint, shear strength

## Abstract

The purpose of the paper is to determine the impact of surface treatment on the strength of adhesive joints, made from various steel sheets. Two variants of the surface treatment steel adherends were used: without the varnish coat and with the varnish coat, using three polymer-based varnishes (a simple, a hybrid, and a gel). Two types of the adhesives were used to prepare the adhesive joints: a single-component cyanoacrylate adhesive and a two-component epoxy adhesive. A strength test of the adhesive joints (EN DIN 1465 standard), a coating adhesion test (ASTM D3359-B standard), and surface topography, as well as surface roughness, parameters (PN-EN ISO 11562, PN-EN ISO 4287, and PN-EN ISO 25178 standards) were used. Based on the strength tests, it was observed that the adhesive joints, with the hybrid varnish onto the adherend’s surface, achieved markedly lower shear strength. The best results, in terms of the adhesive joint strength, made using the cyanoacrylate adhesive were achieved for the joints where the adherends were coated with a simple varnish, while in the joints made using the epoxy adhesive, the highest shear strength was achieved by the joints of sheets whose surfaces were coated with the gel varnish.

## 1. Introduction

An important process, necessary for obtaining adhesive joints with specific properties, is the proper surface treatment. The more carefully the surface is treated, the stronger the adhesive joints will be [1,2]. A high adhesive strength can be achieved if several rules are followed, depending on the type of the adherends, i.e., degreasing, cleaning the surface in an acidic or an alkaline bath, removing unneeded coats from the adherends surface by grinding or degreasing, coating the surface with the primers to produce a new active layer, etching, corona treatment, plasma treatment in order to modify the surface activity, or applying primers [2,3]. This great importance of the surface treatment for the adhesive processes, such as bonding, laminating, and painting, has been described in many works [1,4,5]. During the surface treatment, depending on the method used, the irregularities are formed on the surface of the material, into which adhesive or other adhesive material penetrates, creating the mechanical anchors. Such a joint is characterized by much greater strength than the joints of the materials without such irregularities. That is why it is so important to select a surface preparation method according to the type of material. The importance of creating irregularities was presented in the work by Li et al. [1]. They highlighted that microcraters and nanopores were obtained after using chemical treatment, the cross-sectional interphase matched well and formed the micro-interlocks, and at the nanoscales, the polymer resin had a frictional interaction with the metal adherend during the de-bonding process.

Mamalis et al. [4] investigated the effects of several methods of physical and the chemical treatments on the surface morphology of aluminum alloy sheets and on the adhesive strength at the interface between two adherends, i.e., composite-metal. The obtained results highlighted that the physical and chemical treatments used on the aluminum alloy sheet surface changed the morphology of the adherend surface and the surface energy to variable degrees, which, in turn, successfully enhanced the interfacial bonding force between the adherends. Gao et al. [6] examined the influence of the surface treatment on the bond strength of the aluminum-steel clad strips in cold roll bonding, and they noticed differences in bond strength between applied surface treatments: a belt grinding and a wire brushing. It was noted that the belt grinding treatment, used effectively, removes the various contaminants, such as oxide layers, and shows an adequate degree of surface hardening. Rudawska and Wahab [7] determined the influence of the various coatings (cataphoretic and the powder) and of the application method of the primer, on the adherend’s surface, on the strength results of the adhesive joints of EN AW-5754 aluminum alloy. The usage of the primer meaningfully affects the strength of the adhesive joints of both uncoated and coated EN AW-5754 aluminum alloy sheets.

Many structures use various types of the protective and the protective-decorative coats, including the paint coats. The various types of the coatings both protect the materials against the effects of the aggressive factors [8,9,10,11,12,13,14] and constitute a base (layer) for further adhesive processes (promote the adhesion), including bonding [15,16].

Painting is among the most common ways to protect the metal structures against corrosion. The paint coats are used as protection, but they are also decoration. They provide the surfaces with a specific color and gloss. Two types of the polymer coats are identified. These are the paint coats and the polymer (synthetic) material coats. The paint coats are applied to various types of the surfaces. The primary purpose of applying the coats is a corrosion protection. They are used in virtually every area of everyday life as well as in many industries. The polymer coats are produced by applying the painting materials or the polymer materials. The painting materials are highly complex mixtures and contain varied components, depending on the type of the paint and its purpose. Ecco et al. [9] underlined that the metals are mostly painted to improve the corrosion protection of the coated samples, and the paints can be used to also supply visual aspects, as visual appearance is as important as the corrosion protection. In this work, the color changes of the waterborne paints versus field exposure are investigated. Alexander et al. [16] emphasized that plasma polymers (PP) can be deposited to form highly adherend overlayers, on a range of the metals, to promote adhesion and inhibit corrosion, while also playing an important role in forming a strong interfacial bond with the adhesive. Ozdemir et al. [10] studied the adhesion strength of four wood species coated with cellulosic varnish, and they pointed out that overall higher roughness characteristics of the specimens exhibited enhanced adherence between the varnish and adherend, leading to higher values of the adhesion force. Söğütlü et al. [17] presented the impact of the surface roughness of the wood adherends on the adhesion strength of the varnish layers.

This paper focuses on analyzing the impact of the method of surface treatment on the strength of adhesive joints, made from three types of steel sheets, on the strength of the adhesive joints of these materials, and on what surfaces the painting coats are applied in the form of three different polymer varnishes. Additionally, the method of surface treatment (with or without a coating—reference samples), as well as the type of adhesive used (cyanoacrylate and epoxy adhesives), are both considered. In the article, two types of the adhesive joints are tested: (i) the adhesive joints consisting of uncoated adherends and (ii) the adhesive joints formed from coating materials. The adhesion tests performed allow for determining of the adhesion force between the coating and the steel. The strength tests allow for determining the strength of the entire system for the variant consisting of three and five adhesive bonds.

## 2. Materials and Methods

### 2.1. Adherends

The samples with the following dimensions were used for the adhesive joints: 1.5 × 20 × 100 mm made of a special-purpose alloy spring steel—50CRV4 (PN-EN 10132-4) (VAlfaTech, Koło, Poland), a hot-dip galvanized low-carbon steel DX51DZ (steel grade according to EN 10346) (Alfun, Bruntal, Czech Republic), and a high-alloy acid resistant steel grade 1.4404 (AlfaTech, Koło, Poland), according to PN-EN (X2CrNiMo17-12-2). The mechanical properties of the joined materials are shown in Table 1.

### 2.2. Adhesives

Two types of the adhesives were used to produce the adhesive joints: a cyanoacrylate adhesive and an epoxy adhesive. The 10-second cyanoacrylate adhesive (DRAGON Poland, Kraków, Poland) is a single-component adhesive containing ethyl 2-cyanoacrylate. It is a mixture of ethyl 2-cyanoacrylate and poly(methyl methacrylate). The percentage weight share of ethyl 2-cyanoacrylate is 80–90%, while for poly(methyl methacrylate), it is 1–14%. This adhesive is distinguished by its very fast reaction time, which has led to it being referred to as a one second adhesive. It has the consistency of a thin liquid and is colorless. It has numerous household and industrial applications, particularly owing to its very short curing time. Selected physical properties of the adhesive are shown in Table 2.

The epoxy adhesive is a two-component adhesive containing an epoxy resin (a component A) and a curing agent (a component B), (DRAGON Poland, Kraków, Poland). The component A is a product of the bisphenol A reaction with the epichlorohydrin, and its percentage weight share in the adhesive is 80–95%. The component B is a polyaminoamide-based curing agent, and its percentage weight share in the adhesive is 90–100%, and a triethylenetetramine-based curing agent, whose percentage weight share is 2.5–4.5%. In terms of colors, the component A is a light brown liquid, while the component B is a reddish-brown liquid, and after mixing, the adhesive blend takes on a creamy white color and is a highly viscous liquid with an amine odor. This epoxy adhesive forms hard joints resistant both to water and most solvents. The product is characterized by water resistance, strength, thermal resistance from −20 °C to +100 °C, and a highly viscous consistency. The adhesive cures after 24 h. The mechanical properties of the epoxy adhesive are provided in Table 3.

Adding the curing agent (the component B) to the component A initiates the curing process. Both components need to be added in equal amounts and mixed thoroughly so they form a single whole. An adhesive blend prepared this way remains suitable for use for 30 min. At temperatures below 5 °C, the adhesive does not cure. The curing process is achieved within 4 h. This process can be accelerated by heating the joint with warm air as the higher the ambient temperature, the faster the adhesive cures. After waiting for 24 h, the joint attains its full mechanical strength and can be further processed after this time.

The designations for the adhesives used in the tests are the following:

- Cyanoacrylate adhesive—C;
- Epoxy adhesive—E.

### 2.3. Characteristics of the Surface Treatment Method

The steel sheet samples’ surface treatment was performed in two variants: A and B (Table 4).

#### 2.3.1. Variant A

To compare what effect the application of the varnish on the samples had on the adhesive joint strength, the tests were also performed on the samples made from the same steels as those prepared for the bonding, but they were treated only by degreasing their surface with acetone, which served as reference samples. Then, 20 samples from each type of steel were prepared in this way (Table 5). The degreasing process involved degreasing three times, with acetone, by wiping with the non-dusting pads. After the last degreasing, the surface was left to dry.

#### 2.3.2. Variant B

This variant consists of two stages: the first stage, vibro-abrasive treatment using a working medium in the form of porcelain blocks, and in the second stage, a polymer varnish coat was applied.

Stage 1

Before applying the varnish coat, the samples for bonding in variant B were treated mechanically, using the vibro-abrasive method to achieve a specific roughness of the surface. This operation produced a surface with a repeatable slight roughness without grooves or scratches. The vibro-abrasive treatment was performed using a W50 circular vibrator (Avalon, Nowa Wieś Lęborska, Poland). The principle of operation of this machine involves two composite movements, which are (i) a vibrating movement, which causes vibrations of the work chamber, and (ii) rotating movement, causing a uniform movement of the charge. The samples intended for the coating were placed in the work bowl of the vibration treatment machine, filled with a charge in the form of porcelain blocks (2 × 8 CMP).

Stage 2

Following the vibro-abrasive treatment, the second stage of the surface treatment for bonding involved painting the adherend surfaces with three different polymer varnishes with different chemical bases and curing methods.

Characteristics of the varnishes are provided in Table 6.

**Figure 1:**
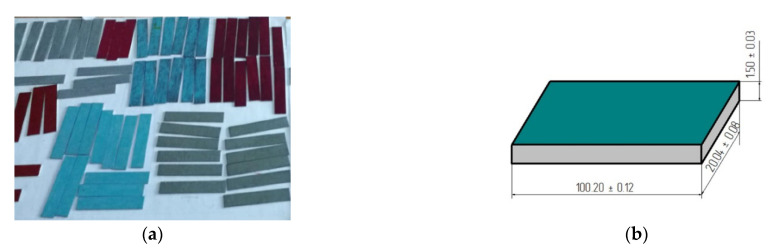
Sample of steel sheets with varnish coats: (**a**) view of samples of steel sheets with coatings; (**b**) scheme and dimensions of a steel sheet sample in mm.

In the case of the hybrid varnish, polymer coat preparation was divided into six stages. This varnish contains three components: base, varnish proper, and external layer. After applying each of these components, curing with a UV lamp was performed. The curing time was 4 days. A single varnish layer was applied to each sample and was left to cure for 3 weeks at a room temperature of 22 ± 2 °C and a humidity of 28 ± 2%. After the three weeks, the adhesive joints were prepared. Figure 1 shows the steel sheet samples after coating.

### 2.4. Adhesive Joints and Bonding Conditions

**Figure 2:**
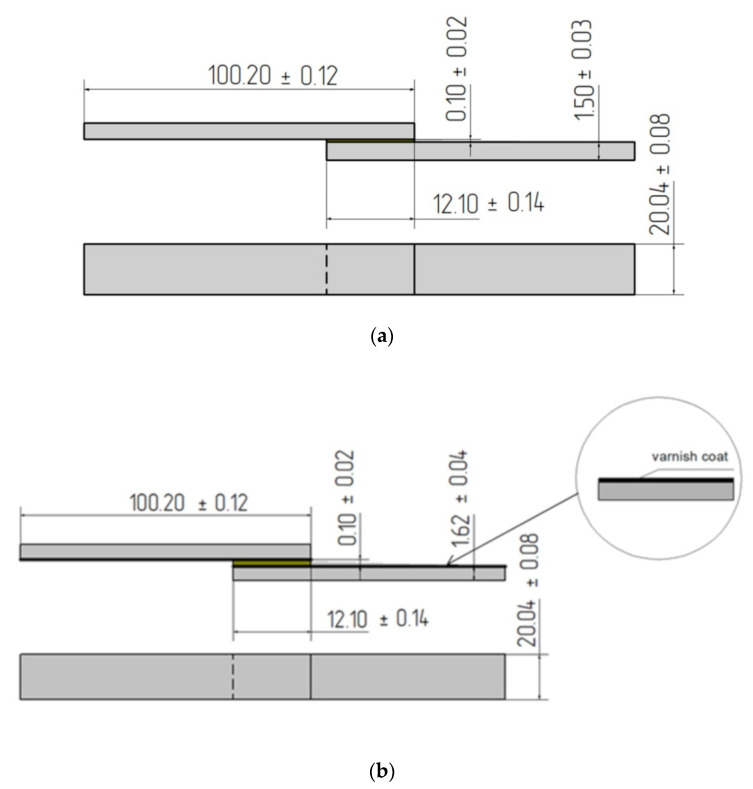
Scheme of single-lap bonded joints samples: (**a**) reference; (**b**) with varnish coat (dimensions in mm).

The single-lap adhesive joints of the structural materials in sheet form, described in Section 2.1, were made in two variants: the reference adhesive joints, without and with the varnish coat, were used for the strength testing. Figure 2 shows a single-lap adhesive joint where the assumed length of the overlap lap for testing was 12.10 ± 0.14 mm, and the assumed adhesive layer thickness was 0.10 ± 0.02 mm.

The shear strength depends strongly on the adhesive layer thickness; therefore, it is important to perform the adhesive joints properly in terms of controlling the adhesive layer thickness of the adhesive joint. In order to obtain the constant adhesive layer thickness, a special device was used to fix the adhesive joints described in the patent [23]. At the same time, six joints were made simultaneously, constituting one series of joints, specified in Table 7. The same adhesive layer thickness was also ensured by the use of the same pressure value during the curing process of the adhesive joint. The theoretical value of the adhesive layer thickness was assumed to be 0.10 mm, while in Figure 2, the actual size of the adhesive layer thickness is shown, and the average value of the adhesive layer thickness is obtained from 60 measurements of the adhesive joints.

All samples used for the testing were assigned arbitrary symbols (Table 4, Table 5 and Table 6). These designations were assigned according to the material type, the surface treatment method, and the type of adhesive used for the joint. Table 7 shows a summary of the adhesive joint samples made of three materials, on which the polymer varnishes were applied, and two types of the adhesives were used, for the surface treatment variant B.

Preparation of the adhesive joints involved several stages. The first stage of the adhesive joint production technology was the surface treatment of the steel adherends, described in Section 2.3. The next bonding operation stage was an adhesive blend preparation. Two adhesive types were used for testing. The first, the epoxy adhesive, was prepared by mixing two components: the curing agent (in itself a mixture of two curing agents—see Section 2.2.) and the epoxy resin at a 1:1 ratio, as recommended by the manufacturer. The components were dosed using a packaging designed specifically for this purpose, a so called blister, which enables proper dosing of the components. Next, the components were mixed using a special spatula, included with the packaging. The mixing time was 30 s. The other adhesive was the cyanoacrylate adhesive, which was a single-component product and did not require any previous preparation. In the case of the epoxy adhesive, the resulting adhesive blend was applied using the previously mentioned spatula. The cyanoacrylate adhesive, however, was applied to the sample surface directly from its tube-shaped packaging, equipped with an applicator. A very important aspect that must be considered when applying an adhesive is to spread the adhesive evenly and to eliminate any air bubbles. Any excess adhesive applied to the length of the overlap needs to be removed. For the cyanoacrylate adhesive, proper precision must be maintained, and the adhesive needs to be applied quickly as its curing time, from the moment of the application to the bonded surface, is only a few seconds. In the case of the epoxy adhesive, the task is easier, as the curing time is fairly long. The adhesives were applied to only one surface of the adherend. The adhesives were prepared at a room temperature of 22 ± 2 °C and a humidity of 28 ± 2%.

The next stage was to position the adherends relative to each other, done using a special bonding device. Subsequently, a pressure of 0.18 MPa was applied to the joints in the lever-equipped retaining device. The pressing lasted for 4 days, which constituted the curing time, after which the load was removed. The curing process was performed for 4 days at a room temperature of 22 ± 2 °C and at a humidity of 28 ± 2%. There were 138 adhesive joints produced in this manner, including 108 adhesive joints where the adherend surfaces were covered with polymer varnish coats and 30 adhesive joints where the adherend surfaces were only degreased with acetone.

### 2.5. Strength Test

The shear strength tests were carried out in accordance with the recommendations of the PN-EN 1465 standard on a Zwick/Roell Z150 testing machine (ZwickRoell GmbH&Co. KG, Ulm, Germany), assuming an initial force value of 5 N and a traverse speed of 5 mm/min.

### 2.6. Coating Adhesion Test (Cross-Cut)

The coating adhesion tests to the substrate were carried out using the incision grid method. The tests were carried out in accordance with the ASTM D3359-B standard with the use of a circular knife equipped with a 6 × 1 mm Elcometer 107 F10713348-6 blade (Ako, Gdynia, Poland). The tests were carried out on the samples covered with the coatings and with the dimensions of 100 × 20 × 2 mm. The cuts were made in the center of the sample, in case of doubts, to the obtained results; subsequent cuts were made in the voids of a given sample. On the basis of the obtained results of the cut lines, the interpretation of the obtained results, presented in Table 8, was possible (according to the ASTM D3359-B standard) [24].

### 2.7. Surface Topography and Surface Roughness Parameters Measurements

The surface topography, and the surface roughness parameters of the steel samples with coatings, were measured with a tracer method with use of the Hommel-Etamic T8000 RC120-400 measuring instrument (JENOPTIC Industrial Metrology Germany GmbH, Schweninngen, Germany). The measurements were conducted with the following standards: PN-EN ISO 11562, PN-EN ISO 4287, and PN-EN ISO 25178. The topographic structure measurements of the examined surfaces consisted of mapping the material profile by passage of the measuring tip on the surface at the speed of 1.20 mm/s. There were 1920 points of measurement set. The adherend surface scanning range was 4.80 mm × 4.80 mm. The following roughness parameters were analyzed: Sa—the arithmetic mean height of the surface, Sz—the maximum height of the surface, Sp—the maximum height of the surface valley, Sv—the maximum height of the surface peak, and Sku—the surface kurtosis.

## 3. Results

### 3.1. Shape and Dimension Correctness

#### 3.1.1. Adhesive Joints Made with the Cyanoacrylate Adhesive

Based on the measurement results, it can be noted that the overlap lengths are very similar, beginning at 11.30 mm and ending at as much as 12.80 mm. In most cases, the overlap length is close to the target value, i.e., 12 mm. The lowest arithmetic mean is 11.88 mm, and the highest is 12.41 mm. Joint thicknesses range from 0.10 mm for adhesive joints, made in samples painted with the gel varnish, made of high-alloy steel, up to 0.15 mm for adhesive joints in spring steel, and coated with the hybrid varnish. The calculated standard deviations and arithmetic means do not indicate any major divergences; the difference between these values is no greater than 0.02 mm.

#### 3.1.2. Adhesive Joints Made with the Epoxy Adhesive

When analyzing the length measurement results of overlaps made with the epoxy adhesive, it can be observed that these lengths are closer to the intended dimension: 12 mm than those for the cyanoacrylate adhesive. This may result from the method of adhesive application, as well as its viscosity. When analyzing the adhesive joints, made with the use of the epoxy adhesive, it should be noted that the arithmetic mean, calculated for the overlap lengths in the case of the three steels, is close to 13 mm. The difference from the intended result is 1 mm. When analyzing the arithmetic means results, calculated for the overlap length, it is visible that these values are very similar and do not exceed the 0.19–0.24 mm range.

### 3.2. Adhesive Joints Strength

To perform a comparative analysis of the influence of the surface state on the adhesive joint strength, the charts were drawn to approximate the average strength values of the tested adhesive joints. The following sample classification criteria were considered when preparing the graphical result interpretations:

- Adherend surface treatment method;
- Adherend material type;
- Adhesive type.

#### 3.2.1. Surface Treatment—Variant A

**Figure 3:**
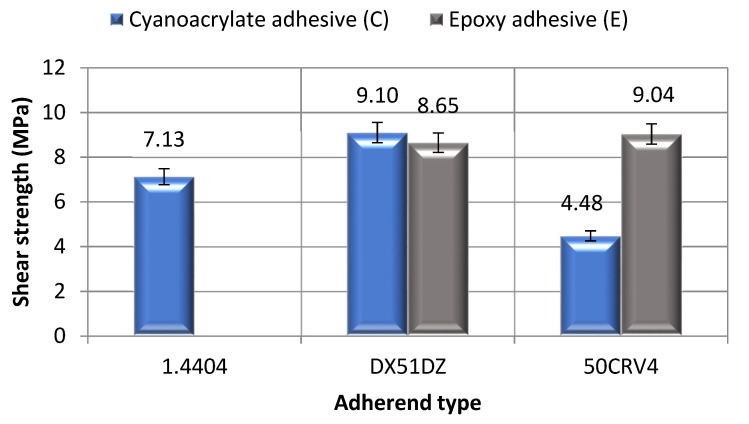
Shear strength of reference sample of adhesive joints: surface treatment—variant A.

Figure 3 shows the average shear strength values for the samples, made of three types of steel, which were bonded using the cyanoacrylate adhesive. The surfaces of the adherends were only degreased with acetone. The comparative analysis does not include the test results for the high-alloy acid resistant steel 1.4404 adhesive joints, bonded with the epoxy adhesive (E-1.4404-A), because the shear strength values, in this case, were so low that the strength testing machine software failed to register them.

The results presented in Figure 3 show that the adhesive joints, made with the low-carbon hot galvanized steel DX51DZ samples bonded using the cyanoacrylate adhesive (C-DX51DZ-A), achieved the highest average shear strength value, specifically 9.1 MPa. The difference between the highest and the lowest average value of the shear strength is over 50%. The lowest shear strength value was recorded for the adhesive joints made with the alloy spring steel 50CRV (C-50CRV-A), measuring at 4.48 MPa. The average shear strength of the high-alloy acid-resistant steel 1.4404 adhesive joints (C-1.4404-A) was 7.13 MPa. The observed differences in the obtained of the strength values can, perhaps, be explained by the different adhesive properties of the adherends. Each material is characterized by a specific value of the surface free energy and, depending on the type of the material, these values can vary significantly. Comyn [25] shows that all metals have oxide coats that are of a high surface free energy, but depending on the type of the layers (depending on the type of the metal substrate), they have different values of surface free energy. This may be one of the reasons for the differences in the obtained values of the strength of the adhesive joints.

**Figure 4:**
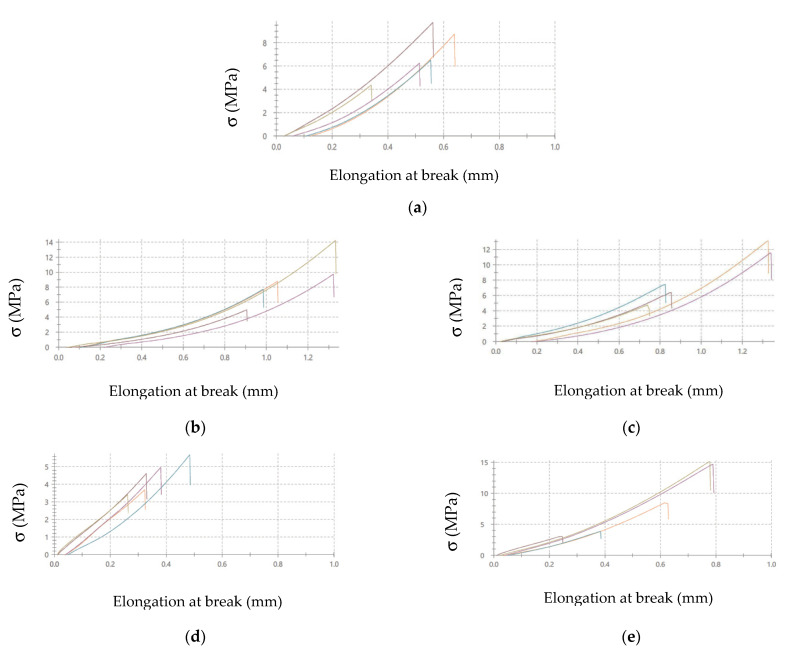
Stress-elongation at break curves of adhesive joints: (**a**) C-1.4404-A, **(b**) C- DX51DZ-A, (**c**) E- DX51DZ-A, (**d**) C-50CRV4-A, (**e**) E-50CRV4-A (designations according to Table 5).

The illustrations of the stress-elongation are shown at break curves in the reference sample of the adhesive joints: the surface treatment—variant A is presented in Figure 4. The applied designations of the adhesive joints are given in Table 5.

It can be noticed that the adhesive joints characterized by the highest strength also have the highest elongation at break value, and it amounts to (the average value): 1.10 mm for C- DX51DZ-A adhesive joints, 1.04 mm for E- DX51DZ-A adhesive joints, and 0.75 for E-50CRV4-A adhesive joints. The smallest elongation at break is obtained for the following adhesive joints: 0.54 mm for C-1.4404-A adhesive joints and 0.38 mm for C-50CRV4-A adhesive joints. It can also be seen that the majority of the adhesive joints made with the epoxy adhesive are characterized by higher elongation at break than those made with the cyanoacrylate adhesive. This may indicate higher elasticity of the epoxy adhesive, which is also somehow noticeable in the obtained curves.

#### 3.2.2. Surface Treatment—Variant B; Adhesive Joints Made with Cyanoacrylate Adhesive

**Figure 5:**
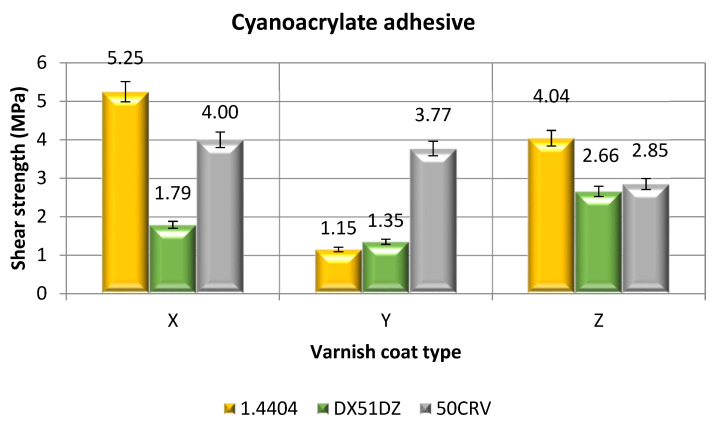
Shear strength of adhesive joints for samples with varnish coats, made with cyanoacrylate adhesive.

Figure 5 shows the shear strength values for the adhesive joints made of three types of the materials, which were bonded using the cyanoacrylate adhesive. Before the adhesive application, the adherend surfaces were coated with a layer of one of three varnishes and, consequently, the charts include three columns, which represent the individual average strength values assigned to specific surface treatment methods.

Regarding the results for 1.4404 steel, it was noted that the lowest average shear strength value during tensioning was exhibited by those joints whose adherends were coated with the hybrid varnish (Y); the average strength value for these joints was 1.79 MPa (Figure 4). The adhesive joints where the sample surface was painted with the simple (X) or gel (Z) varnish have similar values. The highest average shear strength value was observed for the adhesive joints whose adherends were coated with a simple varnish layer; the value was 5.25 MPa. For the adhesive joints whose adherends were coated with the gel varnish layer (Z), the value was 4.04 MPa. The highest standard deviation among the results was observed for the joints made with the adherends whose surfaces were coated with the gel varnish, and the value was 0.44 MPa. The lowest standard deviation value, on the other hand (0.38 MPa), was observed for the adhesive joints of the adherends with the hybrid varnish coating.

When analyzing the shear strength results for the galvanized steel adhesive joints, it can be clearly seen that the lowest average shear strength values were achieved by the adhesive joints whose adherends were coated with the simple varnish (1.15 MPa). The highest average shear strength value was achieved by those joints whose adherends were painted with the gel varnish. The strength value, in this case, was 2.66 MPa. Low value scatter was observed in the test results. The standard deviations for all the tested variants were, for the adhesive joints whose the adherends were coated with a simple varnish layer—0.36 MPa, for the hybrid varnish—0.31 MPa, and for the gel varnish—0.81 MPa.

Analyzing the shear strength results, for the adhesive joints made with alloy spring steel, a low scatter of the average shear strength values among the three surface treatment methods was noted. The lowest values were achieved for the adhesive joints whose adherends were painted with the gel varnish (variant Z) and it amounts to 2.85 MPa. The highest average shear strength value (4.00 MPa) was observed for the adhesive joints where the adherends were coated with the simple varnish layer.

The small standard deviations testify to the high repeatability of the obtained results, which is a result of, inter alia, correct individual technological operations of making the adhesive joints. The standard deviations, in most cases, are less than 10% of the mean value. The bonding technology is classified as a special process, and many factors often have a synergistic effect on the correctness of the adhesive joints. Therefore, it is important to adhere to the technological regimes when making the adhesive joints.

Referring to the results obtained for the adhesive joints, made with the cyanoacrylate adhesive with adherends with simple varnish coatings (X), the difference between the highest and lowest mean shear strength values (Figure 5) was 66%. On the other hand, in relation to the results obtained for the adhesive joints of the adherends with hybrid varnish coatings (Y), the difference between the highest and lowest average shear strength values was nearly 70%. However, for the last group of the adhesive joints, in which the adherends are coated with gel varnish (Z), the difference is 34%. These results may also indicate a high dependence of the type of the adhesive and the type of varnish coating on the adhesive joints strength.

#### 3.2.3. Surface Treatment—Variant B; Adhesive Joints Made with Epoxy Adhesive

**Figure 6:**
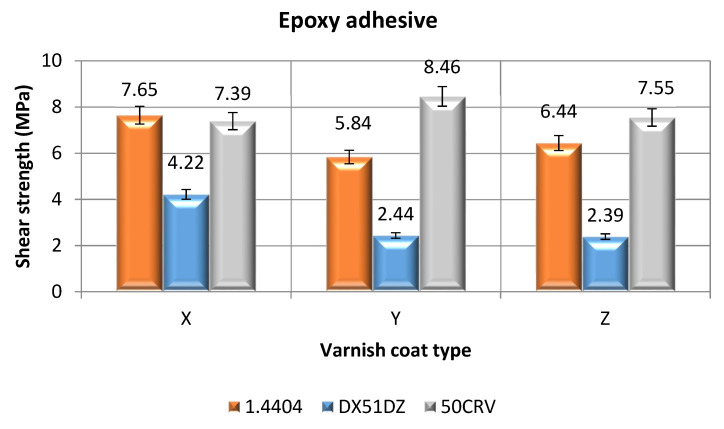
Shear strength of adhesive joints for samples with varnish coats, made with epoxy adhesive.

Figure 6 shows the shear strength values for the adhesive joints, made of three types of materials, which were bonded using the cyanoacrylate adhesive. Before the adhesive application, the adherend surfaces were coated with a layer of one of three varnishes.

Comparing the results shown in Figure 6 for the high-alloy acid-resistant steel joints, it can be clearly noted that the lowest shear strength values were achieved by joints made of adherends whose surfaces were coated with the hybrid varnish (Y). The average shear strength value for this surface treatment variant was 5.84 MPa. The adhesive joints made of the samples, whose surfaces were coated with the simple varnish (X), achieved the highest shear strength value, ranging from 6.94 MPa to 8.36 MPa, with an average of 7.65 MPa.

When analyzing the shear strength results for the galvanized steel adhesive joints, it was observed that the lowest average shear strength values were achieved by the joints whose adherends were coated with the gel varnish (Z)—2.39 MPa. The range of these values is low, as the standard deviation is only 0.08 MPa. The highest shear strength values were clearly achieved by the adhesive joints made of the adherends coated with the simple varnish (X). The average for these values was 4.22 MPa.

Analyzing the shear strength results for the alloy spring steel adhesive joints, it was observed that the lowest shear strength was achieved by the joints where the adherend surfaces were painted with the hybrid varnish (Y)—8.46 MPa. The highest average shear strength value was observed for the adhesive joints made from the adherends painted with the gel varnish (Z); the value is 7.55 MPa.

In relation to the results obtained for the adhesive joints of the adherends with simple varnish coatings (X), the difference between the highest and the lowest average shear strength value (Figure 6) was almost 45%. In relation to the results obtained for the adhesive joints of the adherends with hybrid varnish coatings (Y), the difference between the highest and the lowest average shear strength value was near 70%. However, for the last group of the adhesive joints, in which elements covered with gel varnish (Z) are bonded, the difference is 68%. These results may also prove the high dependence of the type of the adherends and the type of the varnish coating on the strength of the adhesive joints. Moreover, the type of the adhesive (Figure 5 and Figure 6) also affects the strength of the adhesive joints and the various differences between the analyzed variants of the adhesive joints. Therefore, it is important to simultaneously analyze many factors influencing the strength of the adhesive joints.

**Figure 7:**
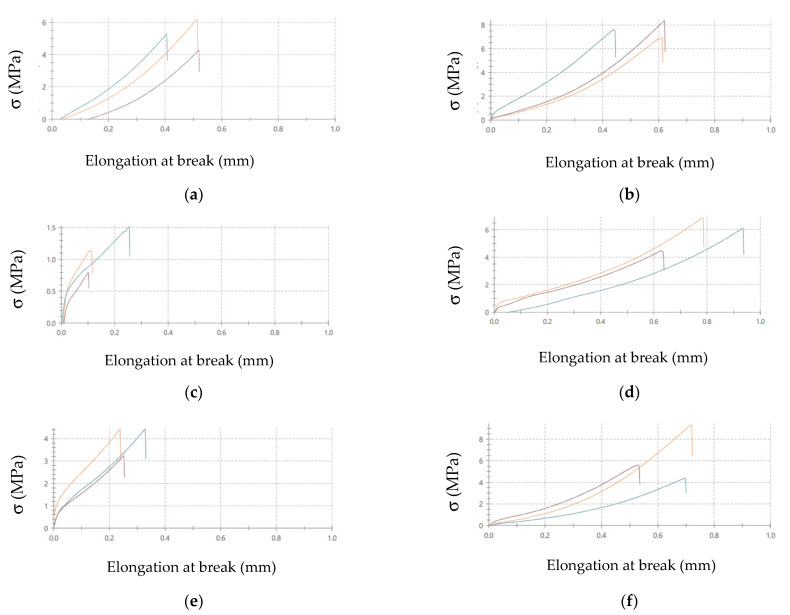
Stress-elongation at break curves of simple varnish (X) coated steel adhesive joints: (**a**) C-1.4404-XB, (**b**) E-1.4404-XB, (**c**) C-DX51DZ-XB, (**d**) E-DX51DZ-XB, (**e**) C-50CRV4-XB, (**f**) E-50CRV4-XB (designation according to Table 7).

The stress-elongation at break curves were also obtained in the strength tests. The example curves of the adhesive joints of simple varnish (X) coated steels, made with two types of the adhesive, are given in Figure 7 for comparison. The adhesive joints designations are given in Table 7.

Similarly to the adhesive joints, for which surface preparation variant A was used (Figure 4), it can be noticed that the adhesive joints made with the epoxy adhesive are characterized by higher elongation at break than those made with the cyanoacrylate adhesive (Figure 7). In most cases, there is a twofold increase in the elongation at break of the analyzed adhesive joints made with the epoxy adhesive compared to the cyanoacrylate adhesive. In the case of the adhesive joints with other coatings, similar relationships were obtained. The elongation at break value may be one of the parameters to consider when designing the adhesive joints.

It should also be mentioned that the shear stress distributions in the single-lap joint specimens are not uniform along the overlap length (~12.10 mm), because strong stress concentrations occur at both ends of the overlap length (3.3 mm). The obtained coating adhesion test results are summarized in Table 9, Table 10 and Table 11.

Based on the interpretation of the results of the cut lines (in accordance with the information in Table 8), it can be assumed that the best properties, in terms of the adhesion of the coating to the substrate, are characterized by those whose results can be classified as group 5B. Analyzing the test results presented in Table 9, Table 10 and Table 11, it can be seen that the best adhesion (defined as a 5B group) is characteristic for the coatings made with the simple varnish (X) on 1.4404 steel and 50CRV steel, while in the case of DX51DZ steel, the hybrid varnish (Y) showed the highest adhesion. The worst adhesion, according to the 3B classification, showed the gel varnish coating (Z) on 50CRV steel and the simple varnish (X) on the low-carbon hot galvanized steel (DX51DZ). In general, it was noticed that gel varnish showed the weakest adhesion to the surface of the analyzed steel sheets, i.e., the two samples were defined as 4B and one as 3B. However, group 3B is worse in terms of adhesion, but it is an acceptable level as it does not delaminate the coating. In this case, as defined in Table 8, total area of damage greater than 5% but not more than 15%.

### 3.3. 3D Surface Topography and Surface Roughness Parameters

Table 12, Table 13, Table 14 and Table 15 present the results obtained during the topographic structure measurement of the adherends (three types of steel), whose surface was subjected to coatings—the surface treatment variant B. Due to the difficulties in the correct performance of measurements, and due to the insufficient hardness of the coating made of the hybrid varnish (Y), the surface topography was not tested.

Based on the results presented in Table 12, Table 13, Table 14 and Table 15, it should be stated that the surfaces of the coatings, made of both the simple varnish and the gel varnish on 1.4404 and 50CRV4 steel sheets, obtained a higher degree of the surface development than the same varnish coatings applied to the surface of DX51DZ steel. This may indicate the influence of not only the type of varnish but also the type of substrate on the surface topography. This aspect was also emphasized in the work of Söğütlü et al. [17] and Ozdemir et al. [11]. The surface kurtosis (Table 14 and Table 15) obtained a positive value in all cases of adherends. It means that the individual results are very close to the arithmetical mean, especially in case of the 1.4404 (ARS) steel for the simple varnish (X) and DX51DZ (HDGS) steel for the gel varnish (Z).

## 4. Discussion

A comparative analysis of the influence of surface treatment on adhesive joint strength, considering the surface treatment variant A (degreasing), performed based on criteria of: three steel types and two adhesive types, demonstrated that, in the case of the adhesive joints made from the high-alloy acid-resistant steel (1.4404) and from low-carbon hot galvanized steel (DX51DZ), prepared using the cyanoacrylate adhesive, the shear strength is higher than for the adhesive joints made of the same materials using the epoxy adhesive.

A comparative analysis of the surface treatment on the adhesive joint strength, performed based on criteria of: three steel types, three varnish types (surface treatment variant B), and two adhesive types, demonstrated that:

- The shear strength of the adhesive joints, made using the epoxy adhesive, reaches higher values than the shear strength of the adhesive joints made using the cyanoacrylate adhesive;
- For the adhesive joints made with the cyanoacrylate adhesive, the highest shear strength is obtained for the adhesive joints whose adherends were made of high-alloy acid-resistant steel (1.4404); for the adhesive joints made using the epoxy adhesive, it was observed that the lowest shear strength values, in every case, were exhibited by those adhesive joints whose adherends were painted with the hybrid varnish; the other analyzed joints had similar values in most cases.

Comparing the surface treatment variants A and B, it was noted that the joints where the adherend surfaces were not coated with the polymer varnish layers (surface treatment variant A) exhibited higher standard deviation values than for joints whose samples were coated with varnishes (surface treatment variant B). Sonmez et al. [12] underlined that the moisture content, the type of wood, and the type of varnish all have significant effect on the adhesion. The conducted research also showed that the type of coating has a large impact on the adhesion, as well as the type of adherend on which the varnish layer was applied. Gao et al. [6] also indicated that the main role of the surface treatment is to purify the surface, and the existence of a hardened layer always has a positive effect on the interfacial bonding.

The research showed that the type of the coating, depending on the type of the substrate, affects the strength of the adhesive joints. The work [7] emphasizes that the type of coatings, depending on the type of pigment (red, white, and black), influences, among others, color stability (i.e., durability) and degradation speed. This emphasizes that research on the influence of the type of coatings on various properties, not only of the coatings themselves but also their various applications, allows for obtaining important information that is necessary when designing various structures. Xie et al. [5] pointed out that the mechanical properties of the bonded joints were always significantly degraded due to the surface oxide scales.

**Figure 8:**
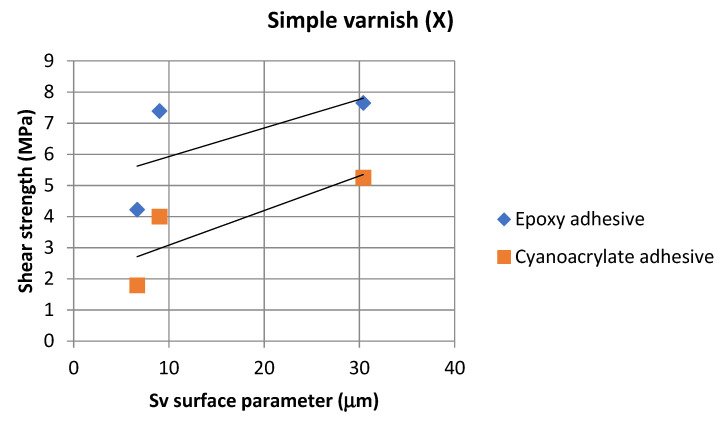
Relations between adhesive joints, shear strength with simple varnish (X), and the Sv surface roughness parameter.

On the basis of the value of the Sv parameter, it can be concluded, about the ability to hold the layer of adhesive materials, by the geometrical structure of the surface. Surfaces that require good wetting should have high Sv values. With reference to the results presented in Table 13 and in Figure 4 and Figure 6, it can be noticed that in the case of the 1.4404 steel, covered with the simple varnish (X), the value of the Sv parameter is the highest and amounts to 30.40 µm. For this material, using variant B of the surface treatment (the varnish coating), the highest values of the adhesive joints strength were also obtained among the analyzed varnishes on this steel: 7.65 MPa in the case of using the epoxy adhesive and 5.25 MPa in the case of using the cyanoacrylate adhesive. The relations between the shear strength of the adhesive joints using variant B of the surface treatment—simple varnish (X)—and the Sv surface roughness parameter was shown in Figure 8.

Based on the results presented in Figure 8, the correlation coefficient (ρ) between the strength of the adhesive joints, in which the adherends were covered with simple varnish (X), made with both types of the adhesives and the Sv surface roughness parameter, was determined. The correlation coefficient was 0.63 for the epoxy adhesive, and for the cyanoacrylate adhesive, it was 0.83, which indicates a strong relationship between these parameters.

**Figure 9:**
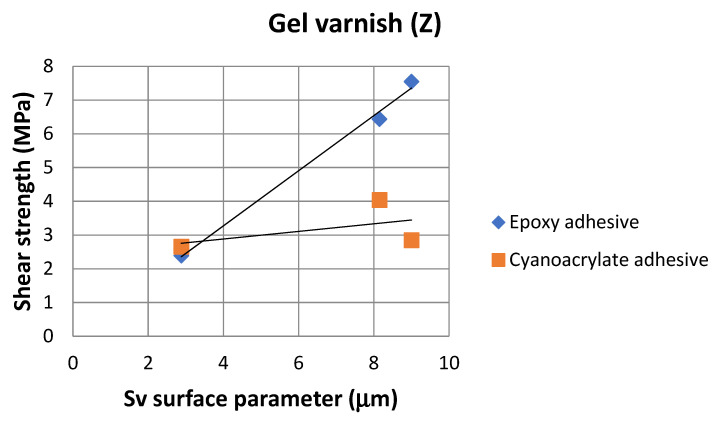
Relations between adhesive joints shear strength with gel varnish (Z) and the Sv surface roughness parameter.

Both in the case of the adhesive joints, made with the cyanoacrylate adhesive (Figure 4), and the epoxy adhesive (Figure 6), it was noticed that the application of the coating made of the gel varnish (Z) was achieved with a higher value of the Sv parameter. The value of the Sv parameter for the surface of the coated sheets is 9.00 μm for the 50CRV4 steel, and for the 1.4404 steel, it is 8.15 μm. The strength of the adhesive joints of these materials with the gel varnish coating (Z) is the highest, respectively: 7.55 MPa and 6.44 MPa for the epoxy adhesive and 2.85 MPa and 4.04 MPa for the cyanoacrylate adhesive. The relations between the shear strength of adhesive joints using variant B of the surface treatment—the gel varnish (X)—and the Sv surface roughness parameter was shown in Figure 9.

When comparing the results presented in Figure 9, it can be seen that the correlation coefficient (ρ) between the adhesive joints strength, in which the adherends were covered with gel varnish (X) made for the epoxy adhesive, and the Sv surface roughness parameter was 0.99. This proves a very strong correlation between the two analyzed quantities. The correlation coefficient between mentioned quantities for the cyanoacrylate adhesive was 0.50, and this shows that the correlation between the two analyzed quantities is moderate, but significant.

Thus, it can be assumed that the Sv parameter may be an initial determinant of certain prediction of the adhesive joints strength. It can also be supposed that a higher value of this parameter will contribute to obtaining the higher strength of the adhesive joints. Söğütlü et al. [17] found that increased surface roughness contributes to the adhesion force of the varnish layer by the existence of chemical and mechanical bonds between the wood surface and the liquid varnish. Adhesive bonds are formed when the varnish liquid fills the irregularities present on the wood surface and then cures. Similar relationships were observed in the case of the adhesive joints of the steel with the varnish coatings and the adhesives. However, it should be noted that the change in the properties of the adhesive joints, with respect to the surface roughness, cannot be explained simply by increased roughness characteristics and associated with the mechanical anchorage formation, which also increases the effectiveness of the real bonding surface. Both the physical and chemical properties of the surfaces of the adherends should be considered simultaneously. This aspect was emphasized in the work of Harris and Beevers [26] and Boutar et al. [27].

After carrying out the strength test, a visual assessment of the failure of the adhesive joints was made using the EN ISO 10365 standard and information presented in [2,25]. It was noted that: (i) the failure mainly concerned the adhesive layer, (ii) in most cases of the adhesive joints of the adherends with varnish coatings, special cohesion failure (SCF, according to EN ISO 10365) was obtained, and these results applied to both types of the adhesives, (iii) in the case of the adhesive joints of the adherends without the varnish coatings, a special cohesion failure (SCF, according to EN ISO 10365) was noticed in the case of the bonding with the epoxy adhesive, and in the case of the adhesive joints made with the cyanoacrylate adhesive, in addition to the special cohesion failure (SCF, according to EN ISO 10365), an adhesion failure (AF, according to EN ISO 10365) also occurred, (iv) the delamination of the varnish coating was not observed. According to the information presented in the work of Adams et al. [2], the adhesive failure of the interface between the adhesive layer and the adherends is one of the most critical types of the failure occurring, in which case care should be taken with the proper surface treatment if such a failure occurs.

By performing a visual analysis of the failure of the adhesive joints, due to the types of failure obtained with the results of the coating adhesion test, and because of the information provided by Adams et al. [2], it was assumed that the failure analysis would be performed in two aspects: (i) failure related to the coating-substrate system and (ii) failure related to the metal substrate and coating-adhesive systems. Based on the adoption of this method of analysis, it can be concluded that:

(i) No delamination of the varnish coating from the metal substrate was observed, as evidenced by the results obtained during the coating adhesion test. In most cases, the coatings are classified as group 5B, which is the group with the greatest adhesion. This may prove the correct development of the technology for the surface treatment of the tested steel sheets prior to coating, as well as the fulfillment of other conditions related to the application of such a coating on the metal substrate. If the results proving good adhesion between the coating and the metal are obtained, then this is the basic starting point for making the adhesive joints of such layered materials (material with a varnish coating). It was in these studies that it was fulfilled. During the analysis of the results of the coating adhesion test, two cases of the weaker adhesion, assessed in the test as 3B, were noticed, the designation of which is described in Table 8. However, it is still acceptable adhesion, which is confirmed by the lack of the delamination of the coating during the tests of the entire adhesive system containing the adhesive joints between the coating and the substrate steel and between the coating and the adhesive;
(ii) Comparing the information contained in the work [2] and in the EN ISO 10365 standard, it can be concluded that due to the fact that:
  - No adhesive failure was observed, referred to as AF—adhesion failure or ACF (P)—adhesion and cohesion failure (with peel), which is related to the failure of the adhesive;
  - No destruction of the substrate has been observed (adherend), i.e., SF—failure of one or both adhrends (substrate failure), CSF—failure of an adherend (cohesive substrate failure), or the third type of destruction concerning the substrate, i.e., p (DF)—failure trough (partial) delamination (delamination failure);
  - The developed technological process, including both the stage of the surface treatment and the stage of applying, curing, and conditioning the adhesive joints, can be considered correct. For this reason, the assessment of the adhesive joints strength is not affected by irregularities resulting from an incorrect technological bonding process.

It can be assumed that such a two-stage analysis, in the case of the bonding materials, with the coatings is important because, already, at the stage of making the adhesive bonds between the coating and the base material, the evaluation of the adhesion between these materials is the basis for further joining processes.

## 5. Conclusions

The obtained results have led to the conclusion that the method of the adherend surface treatment has a large impact on the quality of the resulting adhesive joints as well as on their strength. Based on the comparative analysis, the following conclusions can be drawn:

- It was observed that, in most cases of the adhesive joints, in which the adherends were covered with polymer varnish coatings (surface treatment variant B), lower shear strength values were obtained than for the adhesive joints made of the reference samples (without varnish coatings—surface treatment variant A);
- The adhesive joints, prepared using the epoxy adhesive, achieve higher shearing strength values than those made using the cyanoacrylate adhesive;
- Considering the surface treatment variant that involved the application of the polymer coats, significantly lower shear strength values were achieved by those joints whose adherends were coated with the hybrid varnish. This may be caused by the fact that the hybrid varnish application process comprises several steps and requires using the additional products to cure the varnish coat;
- The best results, in terms of the adhesive joint strength when using the cyanoacrylate adhesive, were achieved for the adhesive joints where the adherends were coated with the simple varnish, while in the adhesive joints made using the epoxy adhesive, the highest shear strength was achieved by the adhesive joints made of the sheets whose surfaces were coated with the gel varnish;
- The best adhesion is characteristic for the coatings made with the simple varnish (X) on 1.4404 steel and 50CRV steel, while in the case of DX51DZ steel, the hybrid varnish (Y) showed the highest adhesion;
- The Sv parameter may be an initial determinant of certain prediction of the adhesive joints strength.

The strength tests demonstrated that the adhesive joints made from the high-alloy acid-resistant steel (1.4404), the low-carbon hot galvanized steel (DX51DZ), and the alloy spring steel (50CRV4), with the use of the cyanoacrylate and the epoxy adhesives, and with their surfaces coated with one of the three polymer varnishes used for the tests (simple, hybrid, gel), exhibited fairly high shear strength values. The results obtained may constitute a basis for applying these solutions in the design of various structures.

## Figures and Tables

**Table 1:** Selected properties of adherends [18,19,20].

Properties	Steel Type (Designation)		
	1.4404	DX51DZ	50CRV4
Tensile strength σ_TS_ (MPa)	500–700	270–500	1100–1300
Yield point σ_Y_ (MPa)	σ_Y 0.2_ min 200	–	≥900
Elongation ψ (%) min.	40	A_80_ 22	≥9
Elastic modulus E (GPa)	200	–	210

**Table 2:** Selected properties of cyanoacrylate adhesive [21].

Properties	Designation	Value
Color	colorless liquid	–
Ether base	ethyl 2-cyanoacrylate	–
Viscosity (at 25 °C)	–	>3 mP·s
Density (at 20 °C)	–	1040–1100 kg/m^3^
Auto-ignition temperature	–	>85 °C
Melting/freezing point	–	20 °C

**Table 3:** Selected properties of epoxy adhesive [22].

Properties	Value
Viscosity (at 25 °C)	>12,000 mP·s
Density (at 20 °C)	1250 kg/m^3^
Compressive strength	90–115 MPa
Bending strength	80–120 MPa

**Table 4:** Variants of adherends surface treatment.

Variant Designation	Description
Variant A	Stage 1: Degreasing with acetone.
Variant B	Stage 1: Vibro-abrasive treatment using a working medium in the form of porcelain blocks.
	Stage 2: Varnish coat application.

**Table 5:** Designations of adhesives joints—variant A of surface treatment.

Adhered Type	Adhesive Type	Samples Amount	Designation
High-alloy acid resistant steel—1.4404	Cyanoacrylate adhesive (C)	10	C-1.4404-A
	Epoxy adhesive (E)	10	E-1.4404-A
Hot-dip galvanized low-carbon steel—DX51DZ	Cyanoacrylate adhesive (C)	10	C-DX51DZ-A
	Epoxy adhesive (E)	10	E-DX51DZ-A
Special-purpose alloy spring steel—50CRV4	Cyanoacrylate adhesive (C)	10	C-50CRV4-A
	Epoxy adhesive (E)	10	E-50CRV4-A

**Table 6:** Description of polymer varnishes used to produce varnish coats on steel samples.

Varnish Type	Designation	Chemical Base	Curing Method
Simple varnish	X	Ethyl Acetate, Butyl Acetate, Nitroce Nitrocellulose, Adipic Acid/Neopentyl Glycol/Trimellitic Anhydride Cipolymer, Acetyl Tributyl Citrate Heptane, Isopropyl Alcohol, Benzophenone-3, Citral, Retinyl Palmitate, Biotin, D&C Violet No. 2 (CI 60725).	Ambient temperature Curing time: 6 min
Hybrid varnish	Y	Butyl Acetate, Ethyl Acetate, Nitrocellulose, Acetyl Tributyl Citrare, Adipic Acis/Neopentyl Glycol/Trimelltic Anhydride Copolymer, Isopropyl Alcohol, Styrene/Acrylates Copolymer, Stearalkonium Bentonite, Silica, Benzophenone-1, Diacetone Alcohol, Trimethylpentanediyl Dibenzoate, Polyethylene, Stearalkonium Hectorite, Phosphoric Acid.	UV curing in three stages
Gel varnish	Z	Urethane Acrylate Oligomer, HEMA, Hydroxycy-clohexyl Phenyl Ketone, Phenyl Bis (2, 4, 6-trimethyl-benzoyl)-phosphine Oxide, (+/−) CI 77891, CI 77491, CI 77510, CI 77289, CI 77492, CI 77266, CI 77742, CI 77019, CI 77891, CI 77499, CI 77861.	Ambient temperature Curing time: 8 min

**Table 7:** Designations of adhesives joints—variant B of surface treatment.

Varnish Type	Adhered Type (Designation)	Adhesive Type	Samples Amount	Adhesive Joints Designation
Simple varnish (X)	1.4404	Cyanoacrylate adhesive (C)	6	C-1.4404-XB
		Epoxy adhesive (E)	6	E-1.4404-XB
	DX51DZ	Cyanoacrylate adhesive (C)	6	C-DX51DZ-XB
		Epoxy adhesive (E)	6	E-DX51DZ-XB
	50CRV4	Cyanoacrylate adhesive (C)	6	C-50CRV4-XB
		Epoxy adhesive (E)	6	E-50CRV4-XB
Hybrid varnish (Y)	1.4404	Cyanoacrylate adhesive (C)	6	C-1.4404-YB
		Epoxy adhesive (E)	6	E-1.4404-YB
	DX51DZ	Cyanoacrylate adhesive (C)	6	C-DX51DZ-YB
		Epoxy adhesive (E)	6	E-DX51DZ-YB
	50CRV4	Cyanoacrylate adhesive (C)	6	C-50CRV4-YB
		Epoxy adhesive (E)	6	E-50CRV4-YB
Gel varnish (Z)	1.4404	Cyanoacrylate adhesive (C)	6	C-1.4404-ZB
		Epoxy adhesive (E)	6	E-1.4404-ZB
	DX51DZ	Cyanoacrylate adhesive (C)	6	C-DX51DZ-ZB
		Epoxy adhesive (E)	6	E-DX51DZ-ZB
	50CRV4	Cyanoacrylate adhesive (C)	6	C-50CRV4-ZB
		Epoxy adhesive (E)	6	E-50CRV4-ZB

**Table 8:** Interpretation of coating adhesion test results (according to the ASTM D3359-B standard)—Method B adhesion rating scale [24].

Rating	Evaluation Criteria
5B	The edges of the cuts are completely smooth; no squares of the mesh skin have been torn off.
4B	Only small flakes of the coating at the edges of the cut grid torn off. No square of the rectangular slit grid was torn off. The total area of the damaged coating is not more than 5%.
3B	Coating peels off with small flakes along the cut line of the net and visible cracks and small pieces of coating peeling between the lines of the net. Total area of damage greater than 5% but not more than 15%.
2B	The coating flakes off along the cuts partially or fully as long ribbons and/or flakes off partially or completely from the squares of the cut grid. Damage area greater than 15% and less than 35%.
1B	The coating flakes off along the cuts in the form of long ribbons and/or flakes off partially or completely from the squares of the score grid. Damage area greater than 35% and less than 65%.
0B	Any degree of detachment of the coating that cannot be classified as 1B.

**Table 9:** Test results for the adhesion measurement of coatings to the substrate—simple varnish (X).

Varnish Type		Simple Varnish (X)	
Surface View	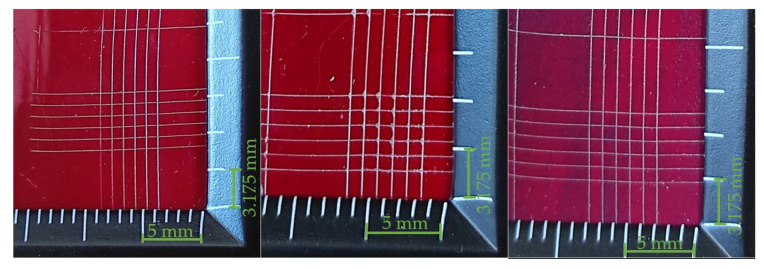		
Adherend (Designation)	1.4404	DX51DZ	50CRV
Coating Adhesion Test Results (According to ASTM D3359-B Standard)	5B	3B	5B

**Table 10:** Test results for the adhesion measurement of coatings to the substrate—hybrid varnish (Y).

Varnish Type		Hybrid Varnish (Y)	
Surface View	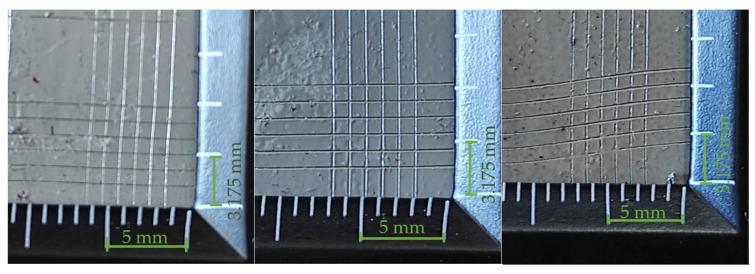		
Adherend (Designation)	1.4404	DX51DZ	50CRV
Coating Adhesion Test Results (According to ASTM D3359-B Standard)	4B	5B	4B

**Table 11:** Test results for the adhesion measurement of coatings to the substrate—gel varnish (Z).

Varnish Type		Gel Varnish (Z)	
Surface View	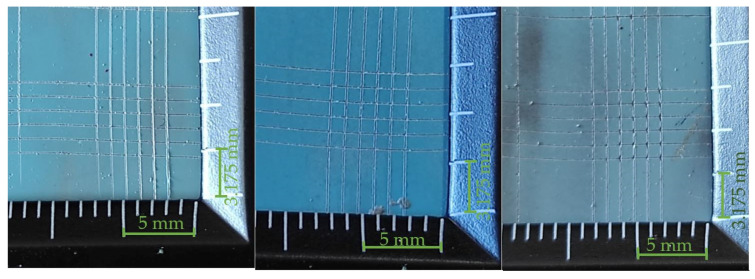		
Adherend (Designation)	1.4404	DX51DZ	50CRV
Coating Adhesion Test Results (According to ASTM D3359-B Standard)	4B	4B	3B

**Table 12:** 3D surface topography for spatial structure adherends with simple varnish (X).

Varnish Type	Simple Varnish (X)
Adherend (Designation)	3D Imagine
1.4404	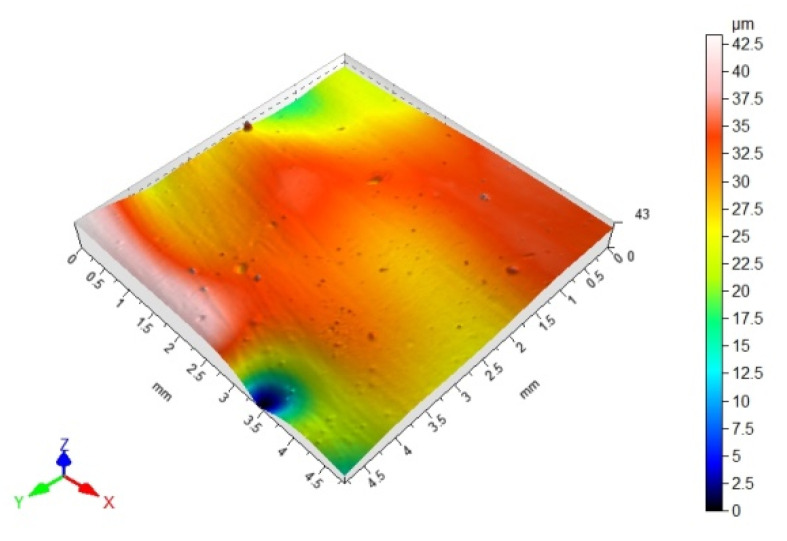
DX51DZ	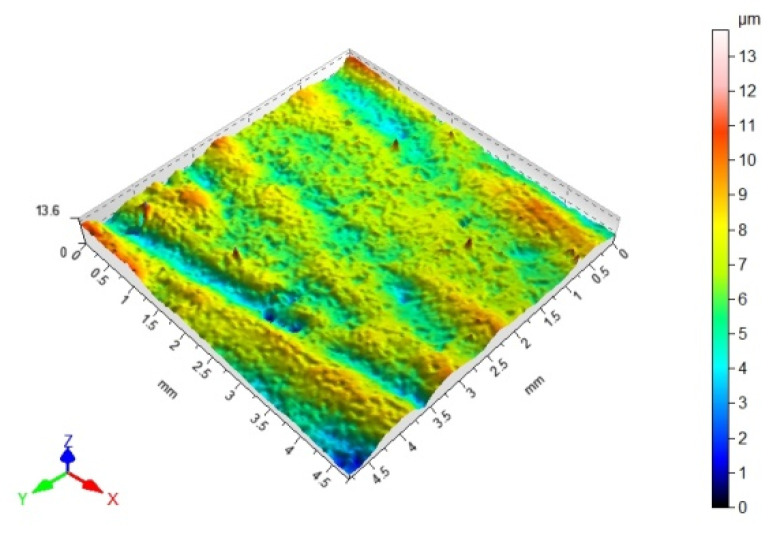
50CRV	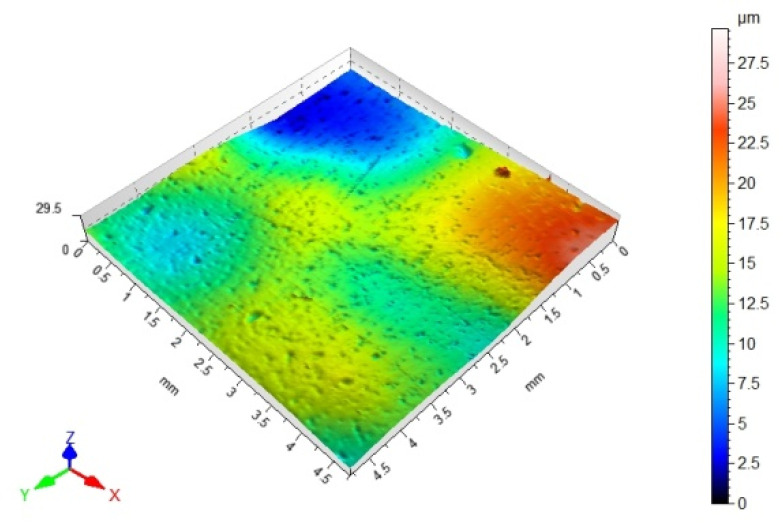

**Table 13:** 3D surface topography for spatial structure adherends with gel varnish (Z).

Varnish Type	Gel Varnish (Z)
Adherend (Designation)	3D Imagine
1.4404	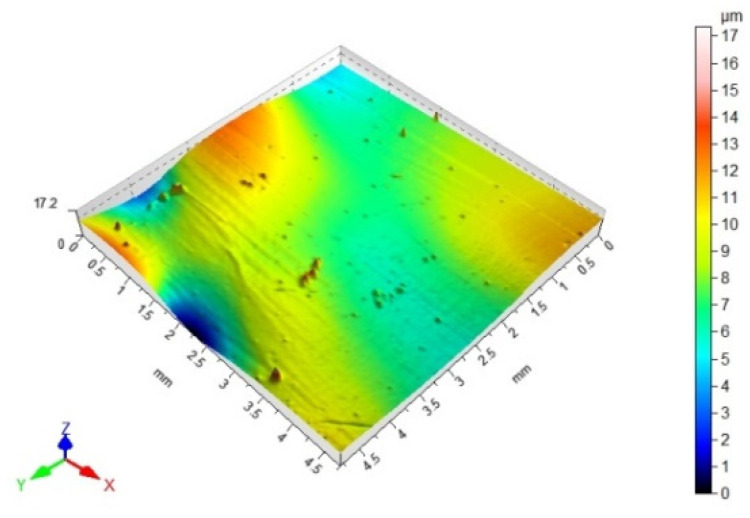
DX51DZ	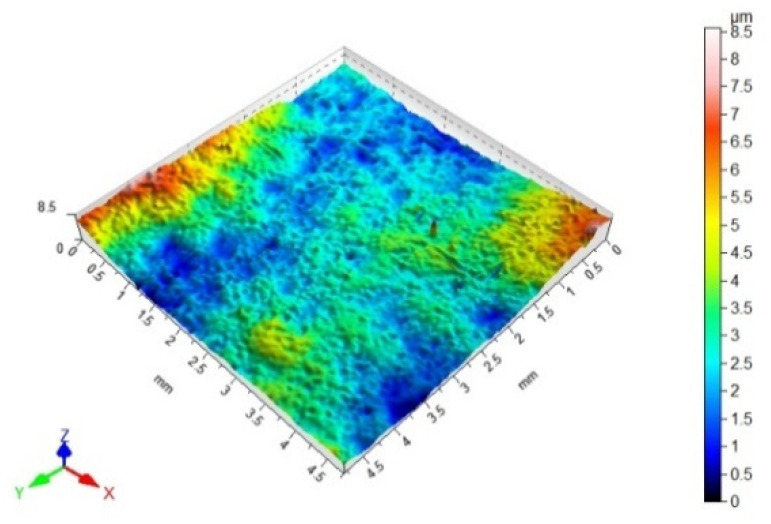
50CRV	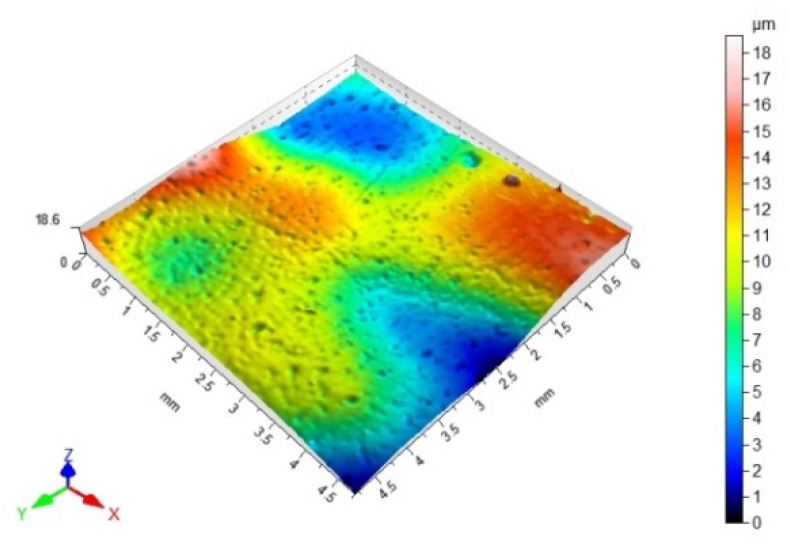

**Table 14:** Average values of 3D height parameters.

Varnish Type	Simple Varnish (X)				
Adherend (Designation)	3D Surface Roughness Parameters				
	Sa (μm)	Sz (μm)	Sp (μm)	Sv (μm)	Sku
1.4404	3.58	43.30	13.00	30.40	6.82
DX51DZ	0.95	13.80	7.09	6.66	3.83
50CRV	2.78	18.70	9.68	9.00	2.24

**Table 15:** Average values of 3D height parameters.

Varnish Type	Gel Varnish (Z)				
Adherend (Designation)	3D Surface Roughness Parameters				
	Sa (μm)	Sz (μm)	Sp (μm)	Sv (μm)	Sku
1.4404	1.48	17.30	9.20	8.15	3.23
DX51DZ	0.89	8.57	5.68	2.88	4.66
50CRV	2.78	18.70	9.68	9.00	2.24

## Data Availability

Data available on request due to restrictions (e.g., privacy or ethical). The data presented in this study are available on request from the corresponding author. The data are not publicly available due to technical or time limitations.
